# Development of a *Cucumis sativus* TILLinG Platform for Forward and Reverse Genetics

**DOI:** 10.1371/journal.pone.0097963

**Published:** 2014-05-16

**Authors:** Adnane Boualem, Sebastien Fleurier, Christelle Troadec, Pascal Audigier, Anish P. K. Kumar, Manash Chatterjee, Abdullah A. Alsadon, Monther T. Sadder, Mahmoud A. Wahb-Allah, Abdullah A. Al-Doss, Abdelhafid Bendahmane

**Affiliations:** 1 INRA-URGV, UMR1165, Unité de Recherche en Génomique Végétale, Saclay Plant Sciences, Evry, France; 2 Bench Bio Pvt Ltd., c/o Jai Research Foundation, Vapi, Gujarat, India; 3 Plant and AgriBiosciences Research Centre (PABC), Botany and Plant Science, National University of Ireland Galway, University Road, Galway, Ireland; 4 Department of Plant Production, College of Food and Agricultural Sciences, King Saud University, Riyadh, Saudi Arabia; Instituto de Biología Molecular y Celular de Plantas, Spain

## Abstract

**Background:**

Cucumber (*Cucumis sativus*) belongs to the *Cucurbitaceae* family that includes more than 800 species. The cucumber genome has been recently sequenced and annotated. Transcriptomics and genome sequencing of many plant genomes are providing information on candidate genes potentially related to agronomically important traits. To accelerate functional characterization of these genes in cucumber we have generated an EMS mutant population that can be used as a TILLinG platform for reverse genetics.

**Principal Findings:**

A population of 3,331 M2 mutant seed families was generated using two EMS concentrations (0.5% and 0.75%). Genomic DNA was extracted from M2 families and eight-fold pooled for mutation detection by ENDO1 nuclease. To assess the quality of the mutant collection, we screened for induced mutations in five genes and identified 26 mutations. The average mutation rate was calculated as 1/1147 Kb giving rise to approximately 320 mutations per genome. We focused our characterization on three missense mutations, G33C, S238F and S249F identified in the *CsACS2* sex determination gene. Protein modeling and crystallography studies predicted that mutation at G^33^ may affect the protein function, whereas mutations at S^238^ and S^249^ may not impair the protein function. As predicted, detailed phenotypic evaluation showed that the S238F and the S249F mutant lines had no sexual phenotype. In contrast, plants homozygous for the G33C mutation showed a complete sexual transition from monoecy to andromonoecy. This result demonstrates that TILLinG is a valuable tool for functional validation of gene function in crops recalcitrant to transgenic transformation.

**Conclusions:**

We have developed a cucumber mutant population that can be used as an efficient reverse genetics tool. The cucumber TILLinG collection as well as the previously described melon TILLinG collection will prove to be a valuable resource for both fundamental research and the identification of agronomically-important genes for crop improvement in cucurbits in general.

## Introduction

Cucumber (*Cucumis sativus*) belongs to the *Cucurbitaceae* family, known as cucurbits and gourds, that includes more than 800 species distributed across tropical and subtropical regions [1]. Besides cucumber, the botanical family *Cucurbitaceae* includes several economically important crops such as melon (*Cucumis melo*), watermelon (*Citrullus lanatus*), squash and pumpkin (*Cucurbita* spp.), bottle gourd (*Lagenaria siceraria*), luffa (*Luffa* spp.), bitter melon (*Momordica charantia*) and others. According to FAO, cucurbits are the third most widely cultivated crops worldwide with a total harvest area of 8 million hectares and a total yield of 194 million tons of vegetables, fruits and seeds annually in 2012 (http://faostat.fao.org). Due to their large production area and economic importance, the last 50 years, cucurbits were subjected to intense breeding programs to improve yield, fruit quality and disease resistance [2]. Cucurbits also served as key model in the field of plant molecular biology and physiology. Cucumber, for instance, is an excellent model for investigating sex determination mechanisms [3]–[6] and vascular fluxes as xylem and phloem saps can be readily collected [7], [8]. Cucumber is a diploid species (2n = 2x = 14) with an estimated genome size of 350 Mb. The availability of the cucumber genome sequence [9] and the accumulation of genetic and genomic resources has encouraged the development of reverse genetic tools to determine and modify gene function. In plants, the most common techniques to produce altered or loss of function mutations are based on insertional mutagenesis [10] and RNA interference [11]. However, because these methods are mainly based on the ability of a given plant to be transformed, their usefulness as general reverse genetics methods is limited to very few plant species and are unconceivable for species recalcitrant to plant transformation, such as cucumber. On the other hand, ethyl methanesulfonate (EMS) mutagenesis is a simple method to saturate a genome with mutations [12]. Targeting Induced Local Lesions in Genomes (TILLinG) combines advantages of random chemical mutagenesis and high throughput mutation discovery methods [13], [14] and generates allelic series of the targeted genes which makes it possible to dissect the function of the protein as well as to investigate the role of lethal genes. Furthermore, TILLinG produces a broad range of mutations including nonsense, missense and splicing mutations which can be used for protein domain annotation. This technique has been successfully applied to a large variety of organisms including plants and animals [15] and has become the method of choice for gene functional analysis in crop species.

In cucumber, sex determination is genetically governed by the genes *Monoecious* (*M*), *gynoecious* (*F*) and *androecious* (a), and the interplay of these three genes can result in a range of sexual phenotypes. Monoecious (*M-ff*) and andromonoecious (*mmff*) individuals bear male flowers and respectively female or hermaphrodite flowers. Gynoecious (*M-F-*) and hermaphrodite (*mmF-*) individuals only bear female and hermaphrodite flowers, respectively [16], [17]. The *androecious* gene (*a*) increases maleness and plants of the *aaff* genotype are androecious bearing only male flowers. In melon, sex determination is genetically governed by the genes *andromonoecious* (*a*) and *gynoecious* (*g*) [18] and plants of (*A-G-*), (*aaG-*), (*AAgg*), and (*aagg*) genotypes are monoecious, andromonoecious, gynoecious and hermaphrodite, respectively. Phenotypically, melon *andromonoecious* (*a*) gene appears to act similarly to cucumber *Monoecious* (*M*) gene. In both species, the dominant allele, *M* in cucumber and *A* in melon, suppresses stamen development in pistillated flowers without affecting male flower formation, whereas the recessive allele, *m* in cucumber and *a* in melon “releases” such inhibition, resulting in bisexual flowers instead of female flowers. In both *Cucumis* species, sexual morphs can be also modified by hormonal and environmental factors, with ethylene playing a major role [19], [20]. Consistent with ethylene being a feminizing agent, we previously demonstrated that the melon *A* gene encodes for the rate-limiting enzyme in ethylene biosynthesis, the 1-aminocyclopropane-1-carboxylic acid synthase, *CmACS*-7 [21]. *CmACS-7* is also specifically expressed in carpel primordia, and in andromonoecious genotypes, a missense mutation leads to loss of enzymatic activity.

In cucumber, using a genetic approach we showed that *CsACS2*, a cucumber ACS highly homologous to *CmACS-7*, co-segregates with the *Monoecious* (*M*) locus. We demonstrated also that *CsACS2,* like *CmACS-7*, is specifically expressed in carpel primordia of buds determined to develop carpels [3]. However, in the absence of transgenic expression data or targeted mutagenesis approach in cucumber, we were not able to clearly conclude that the *M* locus in cucumber encodes for *CsACS2* gene. Here we report the creation of a mutant collection from monoecious cucumber line under controlled conditions and the set up of a TILLinG platform. We validated the quality of the mutagenesis by screening for induced mutations in 5 genes, mainly involved in sex determination and plant architecture processes, and characterized TILLinG lines harboring induced mutations in the *Monoecious* sex gene, *CsACS2*. This work lead to the identification of a missense mutation in *CsACS2* resulting in a sexual transition from monoecy to andromonoecy. Based on this, we concluded that that the *M* gene encodes for *CsACS2*. This result also demonstrates that TILLinG is an efficient tool for functional validation of gene function in crops recalcitrant to transgenic transformation.

## Results

### Production of cucumber Beit Alpha mutant population

The parental cucumber line, Beit Alpha, used to produce the mutant collection is a monoecious line that bears unisexual male and female flowers. The success of the TILLinG approach relies on the construction of high quality mutant libraries. Ideally the mutant population must produce a mutation frequency that is conducive to high-throughput screening but is below a threshold that causes extensive sterility and plant development alteration. To optimize the EMS mutagenesis, we first conducted a ‘kill-curve’ analysis, using a dose range from 0.25% to 1% EMS, on batches of 100 seeds (Table 1). The suitable concentration was determined based on the EMS toxicity at the M1 seed germination. As predicted, we observed that the germination rate was greatly reduced by the incremental increase of EMS concentration (Table 1). At 0.5% EMS concentration, three quarter of the treated seeds germinated, while at 1% EMS, only a quarter of the treated seeds germinated. When the seeds were treated with 0.25% EMS, the germination rate was not affected (Table 1).

**Table 1:** Impact of EMS concentration on seed germination.

EMS dose	M1 plants	Seed germination (%)
0%	100	97
0,25%	100	97
0,50%	100	75
0,75%	100	40
1,00%	100	25

Thus, to produce the cucumber mutant population, the highest EMS doses allowing acceptable seed germination, 0.5% and 0.75% EMS, were retained. Two batches, each of 4,000 seeds, were treated with the selected EMS concentration. Then, the EMS treated seeds were sown in soil and seedlings were grown to fruit maturity in insect-proof plastic tunnels to avoid cross-pollination. Female flowers were hand pollinated with male flowers from the same plants, bagged and the fruits left to develop to maturity. M2 seeds were harvested from individual M1 plants and all the seeds produced by an individual M1 plant were pooled to constitute the corresponding M2 family. From 8,000 treated seeds, 4,660 M2 seed stocks were obtained and used as the basis for the cucumber TILLinG resource.

To assess the quality of the mutagenesis, we investigated the rate of appearance of depigmentation mutants at the cotyledon stage and developmental alterations at young seedling stage. Albino and chlorotic plants, the most frequently observed phenotype in mutagenized populations [12], [22], occurred at the rate of 0.6% of the M2 families. This rate is similar to the rate reported for other well characterized mutant collections [23]–[26]. The most commonly observed developmental phenotypes were related to cotyledon number and morphology, leaf shape and plant architecture. The number of M2 families for each phenotypic category is listed in Table 2.

**Table 2:** Classes of observed mutant phenotypes.

Major category	Subcategory	No. of families
Cotyledon	Color	17
	Shape	13
	Tricotyledon	5
Plant architecture	Multiple meristem	6
	Branching type	17
Leaf	Color	3
	Shape	10

### Setup of cucumber Beit Alpha TILLinG platform

To set up the cucumber TILLinG platform, leaves of individual M2 plants were collected and pooled by families for DNA extraction. Then, DNA samples were prepared from 3,331 M2 families, each representing an independent M1 plant and organized in pools of 8 families as described previously [27].

**Figure 1:**
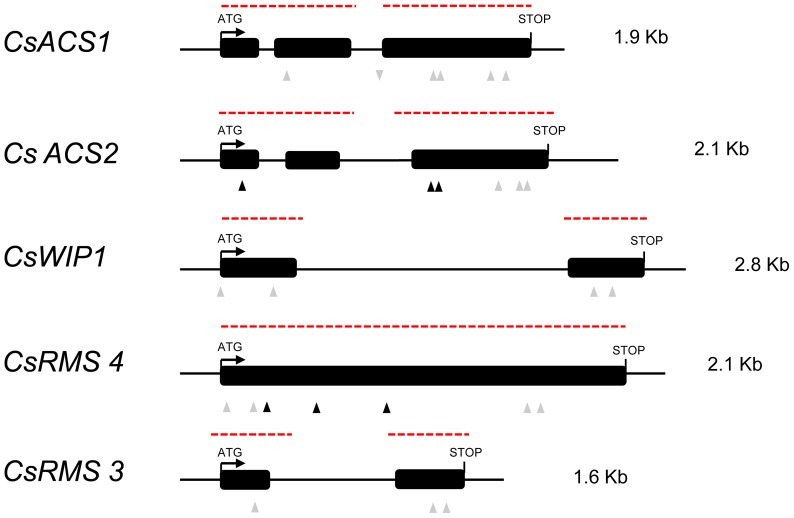
Structure of genes screened for mutations. Black boxes represent the exons. Lanes linking exons indicate introns. Dashed lines in red indicate the genomic regions screened for mutations. Triangles pointing up indicate mutations in coding regions, whereas triangles pointing down indicate mutations in intron. Black and grey triangles represent alterations causing missense and silent mutations, respectively.

To estimate mutation density and validate the cucumber population for future reverse genetics in this species, five genes, *CsACS2*, *CsACS1G*, *CsWIP1*, *CsRMS3* and *CsRMS4*, were screened for induced mutations (Figure 1). *CsACS2*, *CsACS1G* and *CsWIP1* are associated with sex determination. *CsACS2* maps to the *Monoecious* (*M*) locus controlling female to hermaphrodite flower transition [3], [4], *CsACS1-G* maps to the *Female* (*F*) locus controlling plant femaleness [6] and *CsWIP1* is predicted to be orthologous to the melon gynoecy gene, *CmWIP1* [28]. *CsRMS3* and *CsRMS4* are homologous to plant branching genes [29], [30]. *CsRMS4* and CsRMS3 show homology to F-box and α/β-hydrolase proteins, respectively [31]. Mutations in *CsRMS4* and *CsRMS3* are predicted to lead to highly branched plants.

From a total of 8,956 bp fragments screened, 26 induced mutations were identified and their identities determined by sequencing (Table 3). Out of the 26 mutations, 23 were as expected, G/C to A/T transitions [13]. We also observed 3 non-conventional mutations, G/C to T/A, T/A to A/T and T/A to C/G (Table 4), which have also been reported in other TILLinG studies [12], [23], [27], [32]. Sequence analysis of the exonic induced mutations showed that 69.2% were silent, 23% were missense and 7.6% were intronic mutations. Mutation densities varied between 1 mutation every 987 kb, for *CsACS2*, and 1 mutation every 1280 kb for *CsRMS3*. Based on the mutation frequency in the five targeted genes (Table 3), we estimated the overall mutation rate to 1 mutation every 1,147 kb (26 mutations in 8,956 bp of DNA from the 3331 M2 plants screened; Table 3). This mutation density is not significantly higher than the rate of one mutation per megabase seen for TILLinG in barley [33], and is two fold lower than the rate of two mutations per megabase reported for pea, sorghum, tomato and melon [14], [23], [27]. Based on comparison with the mutation frequency observed in other diploid species, 0.5 and 0.75% EMS doses appear to be the adequate doses for TILLinG in cucumber (reviewed in [15]). In summary, we successfully established a cucumber TILLinG platform with sufficient induced mutations per kilobase to enable functional genomic studies in cucumber.

**Table 3:** Tilled genes and mutation frequency in the cucumber mutant population.

Gene name	Amplicon	Identified	Mutation type			Mutation
	size (bp)	Mutants	Missense	Silent	Intronic	density
*CsACS2*	1778	6	3	3	0	1/987 kb
*CsACS1*	2136	6	0	5	1	1/1185 kb
*CsWIP1*	1442	4	0	3	1	1/1200 kb
*CsRMS4*	2447	7	3	4	0	1/1164 kb
*CsRMS3*	1153	3	0	3	0	1/1280 kb
**Total**	**8956**	**26**	**6**	**18**	**2**	**1/1147 kb**

The size of the tilled amplicons, the GC content, the number and type of induced alleles and the mutation frequency per amplicon are shown. The mutation frequency for each amplicon is calculated as follows: {(size of the amplicon) × (total number of samples screened)} / (total number of identified mutants). The average mutation frequency was estimated to be one mutation per 1147 kb.

**Table 4:** Nature of mutations found in the cucumber TILLinG population.

Allele	DNA mutation	AA changed
*CsACS2-1*	G>T	G33C
*CsACS2-2*	C>T	S249F
*CsACS2-3*	G>A	R303R
*CsACS2-4*	G>A	G360G
*CsACS2-5*	G>A	K388K
*CsACS2-6*	C>T	S238F
*CsACS1-1*	C>T	D64D
*CsACS1-2*	C>T	Intron
*CsACS1-3*	G>A	Q337Q
*CsACS1-4*	G>A	K346K
*CsACS1-5*	C>T	L424L
*CsACS1-6*	G>A	S440S
*CsWIP1-1*	C>T	Intron
*CsWIP1-2*	G>A	H90H
*CsWIP1-3*	T>A	A224A
*CsWIP1-4*	C>T	A260A
*CsRMS4-1*	G>A	L9L
*CsRMS4-2*	G>A	P271P
*CsRMS4-3*	G>A	W78C
*CsRMS4-4*	T>C	F100L
*CsRMS4-5*	G>A	N 273 D
*CsRMS4-6*	G>A	L537L
*CsRMS4-7*	G>A	G575G
*CsRMS3-1*	C>T	D152D
*CsRMS3-2*	G>A	P199P
*CsRMS3-3*	G>A	L226L

### Characterization of *CsACS2* induced mutations

Monoecy is characterized by the presence of both male and female flowers on the same plant. In cucumber, this sexual type is controlled by the identity of the alleles at the *M* locus. In melon, we previously showed that the transition from monoecy to andromonoecy results from a mutation in 1-aminocyclopropane-1-carboxylic acid synthase (ACS) gene, *CmACS-7* [3]. To isolate the andromonoecy gene in cucumber, we previously used a candidate gene approach in combination with genetic and biochemical analysis. We demonstrated co-segregation of *CsACS2* gene, the cucumber homolog of *CmACS-7* in melon, with the *M* locus in cucumber [3]. However, in the absence of transgenic expression data or targeted mutagenesis approach in cucumber, we were not able to clearly conclude that the *M* locus in cucumber encodes for *CsACS2* gene. As the cucumber mutant collection described above was created from a monoecious line homozygous for the *M* locus, we took advantage of the cucumber TILLinG platform and screened for induced mutations in the full genomic sequence of *CsACS2*. We identified six mutations among which three were silent and three led to G33C, S238F and S249F missense mutations (Table 4).

**Figure 2:**
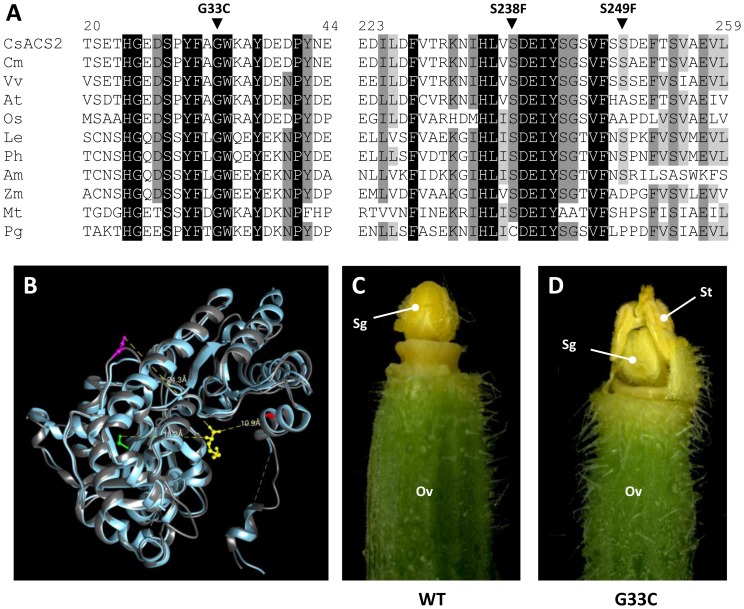
The induced mutation leading to andromonoecy is located in the active site of CsACS2. (**A**) Amino acid alignments of CsACS2 and homologous proteins from *Cucumis melo* (*Cm*), *Vitis vinifera* (*Vv*), *Arabidopsis thaliana* (*At*), *Oryza sativa* (*Os*), *Lycopersicon esculentum* (*Le*), *Petunia hybrida* (*Ph*), *Antirrhinum majus* (*Am*), *Zea mays* (*Zm*), *Medicago truncatula* (*Mt*) and *Picea glauca* (*Pg*). Numbers above the alignment indicate the amino acid positions along the CsACS2 protein. CsACS2 induced missense mutations, G33C, S238F and S249F are shown above the alignment. (**B**) 3D structure model of CsACS2. Superposition of the tomato ACS structure determined by x-ray crystallography [34], indicated in blue and the 3D model of CsACS2 indicated in grey. CsACS2 model was determined using the Geno3D server (http://geno3d-pbil.ibcp.fr). Ball and stick representations show the competitive inhibitor AVG in yellow, and the amino acids G^33^ in red, S^238^ in green and S^249^ in magenta. (**C–D**) Sexual flower types from monoecious (**C**, WT) and the TILLinG andromonoecious mutant (**D**, G33C). The male flowers, identical in all the cucumber types are not shown. Ov, ovary; Sg, stigma; St, stamen.

Among the *CsACS2* induced missense mutations, G33C correspond to one of the natural mutations previously identified [3], [4]. The G33C mutation occurs in a highly conserved amino acid position and was predicted, using the SIFT program, to affect the function of the protein (SIFT score of 0.00) (Figure 2A). Consistent with this prediction, X-ray crystallography analysis located the residue G^33^ in the ACS hydrophobic pocket, where the aminoethoxyvinylglycine (AVG), a structural analog of the S-adenosylmethionine (SAM) substrate, is positioned (Figure 2B, amino acid indicated in red; [34]). Thus the G^33^ amino acid is likely to interact directly with the ACS substrate, SAM and thus G33C mutation should strongly affect the function of the protein. In contrast, the S238F mutation involved a conserved amino acid that is predicted to have lower impact on the protein function (SIFT scores of 0.06; (Figure 2A). Consistent with this prediction, X-ray crystallography analysis located the S^238^ in the β6 strand of the protein away from the active site of the enzyme (Figure 2B, amino acid indicated in green, [34]). The third mutation, S249F, implicated a non-conserved amino acid position and was predicted to not affect the function of the protein, using the SIFT program (score of 0.11) and X-ray crystallography analysis.

To test whether the induced mutations in *CsACS2* affect the cucumber sexual phenotype, we first backcrossed the three mutant lines to the wild type and we followed the segregation of the mutations with the sexual transition in more than 500 F2 plants for each cross. Consistent with the crystallography analysis, the S238F and S249F mutations predicted to not affect the function of the protein had no impact on the sexual type of the plant. In contrast, plants homozygous for G33C mutation showed a sexual transition from monoecious to andromonoecious and as predicted the female flowers were transformed to hermaphrodite. Based on the TILLinG experiment and our previous genetic dissection of the *M* locus [3], we concluded that the *M* locus encodes for *CsACS2* (Figure 2C–D). In the G33C homozygote mutant, the hermaphrodite flowers result from the release of stamen development in carpel-bearing buds.

## Discussion

With the increasing skepticism against transgenic technology and growing transgenic crops, TILLinG, based on chemically induced mutagenesis, has become the method of choice for detailed gene functional characterization and mutation breeding for crop improvement as it yields a range of alleles with different phenotypic strengths. The identification of the optimal dose of a chemical mutagen that maximizes the mutation frequency with acceptable plant viability is a key factor for the establishment of a good TILLinG population. To set up the cucumber TILLinG platform, we first performed a preliminary “kill curve” analysis and observed a strong correlation between the EMS dose and the seed germination rate. To maximize the genome mutation load and the plant survival, two EMS doses, 0.5% and 0.75%, were used to develop a reference EMS mutant collection under controlled conditions. Mutagenesis efficiency was assessed by scoring the occurrence of chlorotic and albino phenotypes. The observed rate of 0.6% of chlorotic and albino phenotypes in the mutant collection is in a similar range of previously described mutant collections and confirms the quality of the mutagenesis. [23]–[26], [35]. To validate the cucumber mutant collection, we screened for mutations in five genes and identified 26 independent alleles. As reported in other TILLinG studies, the EMS mutational specificity shows a strong preference for G/C to A/T transitions, 70 to 99% of the induced mutations [36], [37]. In our cucumber mutant population, most induced mutations were as expected, G/C to A/T transitions, with the exception of the three following mutations, G/C to T/A, T/A to A/T and T/A to C/G (Table 4). The spectrum of observed nucleotide changes is similar to the mutation spectrum observed in rice or tomato [32], [36]. Based on the TILLinG screens, we estimated the overall mutation density to one mutation every 1147 kb with an average of 5 alleles per gene. This mutation frequency is two fold lower than the rate reported for the closest cucurbit *Cucumis melo* [23], for tomato [27], [32] and for sunflower [35] and equivalent to the rate of one mutation per megabase reported for barley [33].

How the gender of a flower or a plant is determined is an important issue in plant developmental biology. Understanding this process also has practical applications in agriculture and plant breeding, as the gender of a flower or plant often limits how the plant is bred and cultivated. In cucumber, sex determination is genetically controlled by three master genes [16], [17]. We previously showed that the *Monoecious* (*M*) locus in cucumber, is likely to encode for *CsACS2* [3], [4], [38]. To test this hypothesis, we screened for induced mutations in *CsACS2*. Six independent mutations were identified and the mutant lines were backcrossed to the wild type and phenotyped. Detailed phenotypic characterization of the TILLinG mutants confirmed that *Monoecious* (*M*) locus encodes for *CsACS2* (Figure 2D). Interestingly, the only mutation, G33C, leading to sexual transition correspond to one of the natural mutations previously identified [3], [4]. This mutation is unlikely to be a contamination, as the G33C mutation was carried by the genetic background of Beit Alpha variety and the mutagenesis was carried out in controlled conditions. One explanation is that some genome sites are more susceptible to mutagenesis. Different studies reported biases of the EMS-induced mutation sites [39]–[41]. The precise reason for the high mutability of specific sites is still unknown. However, we can speculate that highly exposed and not protected DNA sequences could be an easy target for guanine alkylation. Reduced DNA repair at certain sites could also leads to mutation hotspots.

In conclusion, we have developed a reference EMS mutant collection and set up the cucumber TILLinG platform successfully. Through the TILLinG approach, we screened for induced mutations in the *Monoecious* sex determination gene, *CsACS2* and showed that the G33C mutation leads to monoecy to andromonoecy sex transition. Cucumber is also an important model plant in many key areas of plant research, including fruit maturation [42], [43] and the investigation of vascular trafficking of molecules [7], [8]. Hence, by making the cucumber TILLinG platform available for the scientific community, we hope to fulfill the expectations of both breeders and scientists who are using cucumber as plant model.

## Materials and Methods

### Plant Material and EMS treatment

Experiments were carried out using the cucumber, *Cucumis sativus,* cultivar Beit Alpha, a monoecious cultivar. Mature seeds from Beit Alpha cultivar were mutagenized with EMS as described in [23]. Treated seeds were sown in soil and grown under insect-proof plastic tunnels according to standard cucumber cultivation agronomic practices. Mature M2 seeds were collected from individual M1 plants and stored.

### Genomic DNA extraction and pooling

Ten seeds per M2 families were sown in small pots inside the glasshouse. Eight cucumber leaf discs (diameter 10 mm) were collected in 96-well plates containing 2 steel beads (4 mm) per well, and tissues were ground using a bead mill. Genomic DNA was isolated using the DNeasy 96 Plant Kit (Qiagen, Hilden, Germany). All genomic DNA was quantified on a 0.8% agarose gel using λ DNA (Invitrogen, Carlsbad, CA, USA) as a concentration reference and normalized to 40 ng/µl. DNA samples were diluted tenfold and pooled eightfold in a 96-well format.

### PCR amplification and mutation detection

PCR amplification was based on nested-PCR and universal primers. The first PCR amplification is a standard PCR reaction using target-specific primers (Table S1) and 4 ng of cucumber genomic DNA. One microlitre of the first PCR served as a template for the second nested PCR amplification, using combination of specific primers carrying M13 tail and M13 universal primers, M13F700 and M13R800, labelled at the 5′end with infra-red dyes IRD700 and IRD800 (LI-COR, Lincoln, Nebraska, USA), respectively. Mutation detection was carried out as previously described [12]. The identity of the mutations was determined by sequencing. TILLinG request should be addressed to the corresponding author.

### Sequence analysis tools

PARSESNP (Project Aligned Related Sequences and Evaluate SNPs, http://www.proweb.org/parsesnp/) was used to illustrate the distribution of mutations within the gene, and to indicate the nature of each single mutation.

SIFT (Sorting Intolerant from Tolerant, http://sift.jcvi.org/www/SIFT_seq_submit2.html) was used to predict the impact of the mutation on the protein.

Multiple sequence alignment of full-length protein sequences was performed using ClustalW (http://www.ebi.ac.uk/Tools/clustalw2)

### Protein structure modeling

The CsACS2 three-dimensional structures were generated using the Geno3D server (http://geno3d-pbil.ibcp.fr). Superposition of the tomato ACS structure (1IAY.pdb) determined by x-ray crystallography [34], and our three models of CsACS2, was carried out and visualized using the Chimera server (http://www.cgl.ucsf.edu/chimera).

## Supporting Information

Table S1 **Primers used in this study.** Asterisk indicate that the primer is labeled with infra-red dyes IRD700 (forward primers) and IRD800 (reverse primers).
(XLS)
